# Supplementary material for: Supramolecular Enzymatic Labeling for Aptamer Switch-Based Electrochemical Biosensor
Source: Biosensors (Basel). 2022 Jul 12;12(7):514. doi: 10.3390/bios12070514 (PMC9313153; doi:10.3390/bios12070514)
Supplement: Supplementary file 1 [file biosensors-12-00514-s001.zip › biosensors-1747537-supplementary.pdf]

Supplementary Information

Supramolecular enzymatic labeling for aptamer switch-based electrochemical biosensor

Anabel Villalonga[1], Concepción Parrado[1], Raúl Díaz[1], Alfredo Sánchez[1], Beatriz Mayol[1], Paloma Martínez-Ruíz[1], Diana Vilela[1]* and Reynaldo Villalonga[1]*

[1] Nanosensors and Nanomachines Group, Department of Analytical Chemistry Faculty of Chemistry, Complutense University of Madrid, 28040 Madrid, Spain
* Correspondence: divilela@ucm.es, rvillalonga@quim.ucm.es

Table S1. Optimization of the experimental conditions for the assembly and use of the amperometric aptasensor for CEA.

Variable	Range Tested	Selected Value
Apt-ADA concentration (µM)	0 – 60	50
Immobilization time for Apt-ADA (min)	5 – 90	60
Incubation time for CEA (min)	5 – 90	30
HRP-CD concentration (µg/mL)	0 – 200	100
Incubation time for HRP-CD (min)	5 – 120	60
Working pH	7.0 – 7.8	7.4
Working potential (V)	-0.3 – 0.0	-0.2

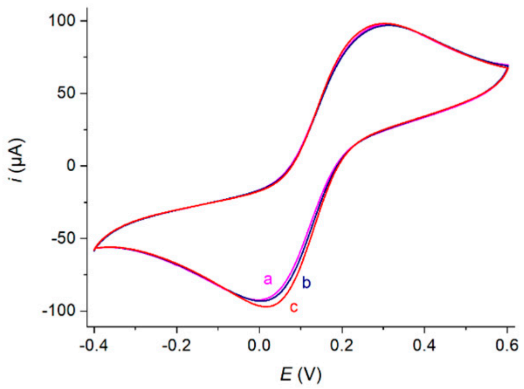

Figure S1. Cyclic voltammograms for Apt-ADA/AuNF/GO/SPE before (a) and after sequential incubation with 20 pg/mL CEA (b) and HRP-CD (c), measured in 0.1 M KCl solution containing 5 mM $K_3[Fe(CN)_6]/K_4[Fe(CN)_6]$ (1:1). Scan rate = 50 mV/s.

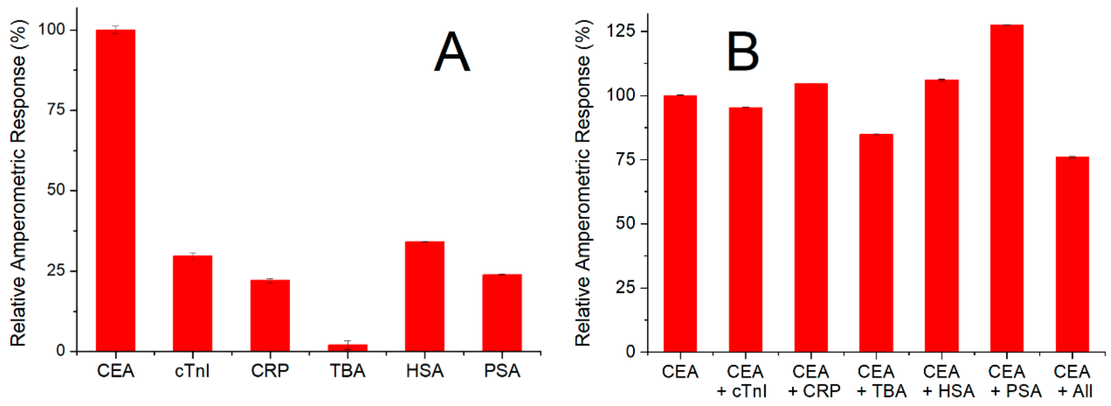

Figure S2. A) Relative amperometric response of the aptasensor toward CEA (2 ng/mL), cardiac troponin I (cTnI, 1 ng/mL), C-reactive protein (CRP, 2 μg/mL), thrombin (TBA, 1.4 ng/mL), human serum albumin (HSA, 50 mg/mL) and prostate specific antigen (PSA, 4 ng/mL). B) Response of the aptasensor toward CEA and mixtures with other potential interfering proteins, at the same concentration cited above. Samples were 1000-fold diluted before measurement.

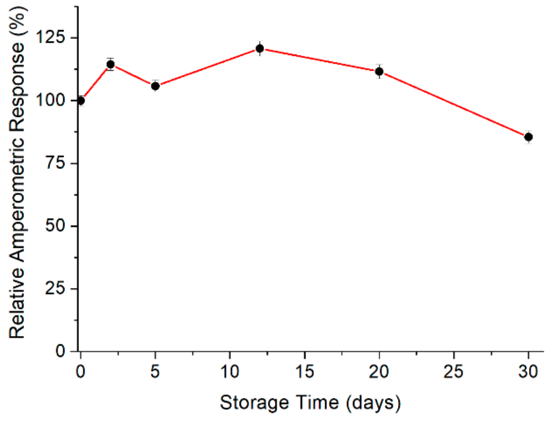

Figure S3. Relative amperometric response of the aptasensor toward 20 pg/mL CEA vs time of storage at 4ºC in dry conditions.

Table S2. Electrochemical Biosensors for the detection of CEA.

Sensing Platform	Detection Method	LOD (g/mL)	Linear Range (g/mL)	Sample Analyzed	Ref.
Apt-Graphene-PEDOT:PSS/**Paper**	EIS	0.45×10^{-9}	0.77×10^{-9}-1.4×10^{-8}	Buffer	1
		1.06×10^{-9}		Human Serum	
BSA/NH_2-Apt/Au@PDA@Fe-MOF/**GCE**	DPV	3.3×10^{-16}	1×10^{-15}-1×10^{-6}	Buffer	2
		-	-	Human Serum	
Aptamer-AuNCs/CEA/ConA@Cusilicate @Fe_3O_4@NCMTs/**AuE**	EIS	5.38×10^{-12}	3×10^{-11}-6×10^{-9}	Buffer	3
		-	-	Human Serum	
Apt/MCH/AuNPs/**PEE-PPy film**	EIS	3.3×10^{-11}	1×10^{-10}-1×10^{-6}	Buffer	4
		-	-	Fetal Bovine Serum	
Strep-Apt-Au/**SPE** Av-MB	DPV	2.8×10^{-13}	1×10^{-12}-1×10^{-7}	Buffer	5
		5.1×10^{-13}	-	Human Serum	
S1/MXC-Fe_3O_4-Ru/**AuE** S2-fc	DPV	4.3×10^{-13}	1×10^{-12}-1×10^{-6}	Buffer	6
		-	-	Clinical Serum	
Ab/AuNPs-Thi-G/**SPCE**	DPV	1×10^{-11}	5×10^{-11}-5×10^{-7}	Buffer	7
		-	1.75×10^{-9}-6.42×10^{-8}	Human Serum	
Ab1-AgNPs-rGO/**SPE** Ab2-HRP	DPV	3.5×10^{-9}	5×10^{-8}-4×10^{-7}	Buffer	8
Apt-ADA/AuNF/GO/**SPE** CD-HRP	Amperometry	-	-	Buffer	This work
		3.1×10^{-12}	1×10^{-11}-1×10^{-9}	Human Serum	

Apt: CEA-Aptamer, PEDOT:PSS:poly(3,4-ethylenedioxythiophene) polystyrene sulfonate, BSA: Bovine Serum Albumin, PDA: Polydopamine, MOF: Metal Organic Framework, GCE: Glassy Carbon Electrode, NCNTs: Magnetic carbon microtubes, AuNCs: AuNPs, AuE: Gold electrode, MCH: 6-Mercapto-1-hexanol, PEE-PPy: pentaerythritol ethoxylate-Polypyrrole, Strep: Streptavidin, Av: Avidin, MB: Methylene Blue, SPE: Screen Printed Electrodes, SPCE: Screen Printed Carbon Electrodes, S1: CEA-DNA1 probe, S2: CEA-DNA2 probe, MXC: 2D Mxene, fc: ferrocene, Ab: CEA-Antibody, AuNPs: Gold nanoparticles, Thi: Thionine, G: Graphene, AgNPs: Silver nanoparticles, rGO: Reduced Graphene oxide, HRP: Horseradish peroxidase, ADA: Adamantane, AuNF:fern-like gold nanoparticles, CD: Cyclodextrin.

References

1. Yen, Y.-K.; Chao, C.-H.; and Yeh, Y.-S.; A Graphene-PEDOT:PSS Modified Paper-Based Aptasensor for Electrochemical Impedance Spectroscopy Detection of Tumor Marker. *Sensors* **2020**, 20(5), 1372. https://doi.org/10.3390/s20051372.
2. Li, J.; Liu, L.; Ai, Y.; Liu, Y.; Sun, H.; Liang, Q.; Self-Polymerized Dopamine-Decorated Au NPs and Coordinated with Fe-MOF as a Dual Binding Sites and Dual Signal-Amplifying Electrochemical Aptasensor for the Detection of CEA. *ACS Appl. Mater. Interfaces* **2020**, 12, 5500–5510. https://doi.org/10.1021/acsami.9b19161
3. Song, J.; Teng, H.; Xu, Z.; Liu, N.; Xu, L.; Liu, L.; Gao, F.; Luo, X.; Free-standing electrochemical biosensor for carcinoembryonic antigen detection based on highly stable and flexible conducting polypyrrole nanocomposite. *Microchim. Acta* **2021**, 188, 217. https://doi.org/10.1007/s00604-021-04859-1
4. Zheng, J.; Wang, Z.; Song, D.; Song J.; Xu; Zhang, M.; Electrochemical Aptasensor of Carcinoembryonic Antigen Based on Concanavalin A-Functionalized Magnetic Copper Silicate Carbon Microtubes and Gold-Nanocluster-Assisted Signal Amplification. *ACS Appl. Nano Mater.* **2020**, 3, 4, 3449–3458. https://doi.org/10.1021/acsanm.0c00194
5. Jimenez-Falcao, S.; Parra-Nieto, J.; Pérez-Cuadrado, H.; Martínez-Máñez, R.; Martínez-Ruiz, P.; Villalonga, R.; Avidin-gated mesoporous silica nanoparticles for signal amplification in electrochemical biosensor, *Electrochem. Comm.* **2019**, 108, 106556. https://doi.org/10.1016/j.elecom.2019.106556

6. Yang, H.; Xu, Y.; Hou, Q.; Xu, Q; Ding. C.; Magnetic antifouling material based ratiometric electrochemical biosensor for the accurate detection of CEA in clinical serum. *Biosens. Bioelectron.* **2022**, 208, 114216. https://doi.org/10.1016/j.bios.2022.114216
7. Wang, Y.; Xu, H.; Luo, J.; Liu, J.; Wang, L.; Fan, Y.; Yan, S.; Yang, Y.; Cai X.; A novel label-free microfluidic paper-based immunosensor for highly sensitive electrochemical detection of carcinoembryonic antigen. *Biosens. Bioelectron.* **2016**, 83, 319-326. https://doi.org/10.1016/j.bios.2016.04.062
8. Lee, S. X.; Lim, H. N.; Ibrahim, I.; Jamil, A.; Pandikumar, A.; Huang, N. M.; Horseradish peroxidase-labeled silver/reduced graphene oxide thin film-modified screen-printed electrode for detection of carcinoembryonic antigen. *Biosens. Bioelectron.* **2017**, 89, 673-680. https://doi.org/10.1016/j.bios.2015.12.030
